# Making predictions from complex longitudinal data, with application to planning monitoring intervals in a national screening programme

**DOI:** 10.1111/j.1467-985X.2011.01005.x

**Published:** 2012-04

**Authors:** M J Sweeting, S G Thompson

**Affiliations:** Medical Research Council Biostatistics UnitCambridge, UK

**Keywords:** Abdominal aortic aneurysm, Hierarchical model, Monitoring intervals, National screening, Prediction, Simulation

## Abstract

When biological or physiological variables change over time, we are often interested in making predictions either of future measurements or of the time taken to reach some threshold value. On the basis of longitudinal data for multiple individuals, we develop Bayesian hierarchical models for making these predictions together with their associated uncertainty. Particular aspects addressed, which include some novel components, are handling curvature in individuals’ trends over time, making predictions for both underlying and measured levels, making predictions from a single baseline measurement, making predictions from a series of measurements, allowing flexibility in the error and random-effects distributions, and including covariates. In the context of data on the expansion of abdominal aortic aneurysms over time, where reaching a certain threshold leads to referral for surgery, we discuss the practical application of these models to the planning of monitoring intervals in a national screening programme. Prediction of the time to reach a threshold was too imprecise to be practically useful, and we focus instead on limiting the probability of exceeding the threshold after given time intervals. Although more complex models can be shown to fit the data better, we find that relatively simple models seem to be adequate for planning monitoring intervals.

## 1. Introduction

Interest often lies in constructing models not only for estimation of the characteristics of a longitudinal process but also for prediction of how the process will evolve in the future. The focus of such predictions can take various forms: a measurement of the process at a given time in the future, the time taken to reach a certain threshold or a probability statement about exceeding a given level at a future time. In this paper these issues are discussed in the particular context of modelling the growth of abdominal aortic aneurysms (AAAs).

AAAs are swellings in the main artery from the heart, defined as an aortic diameter of 30 mm or more, which can be detected and measured by ultrasound scanning. Aneurysms that grow too large are at a substantial risk of rupture, which carries with it a high fatality rate (Bown *et al*., 2002); AAAs are responsible for around 2% of all deaths in men aged over 65 years (Office for National Statistics, 2000). It is now established practice to offer surgery if an aneurysm becomes too large and before rupture occurs, typically when the diameter of the aneurysm is greater than or equal to 55 mm. National screening programmes have recently been established, in the UK and elsewhere (UK National Screening Committee, 2007), where men aged 65 years are invited for ultrasound screening. Within such programmes monitoring intervals need to be determined to limit the probability of exceeding the 55 mm threshold before the next scan. Clearly the length of the monitoring interval should depend on an individual's current AAA diameter but may also be tailored to depend on other patient characteristics associated with rates of growth.

**Fig. 1:**
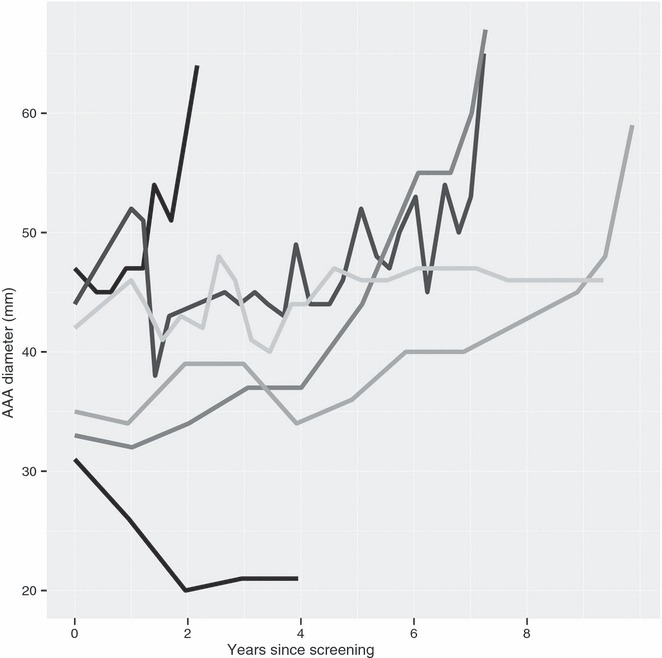
AAA growth trajectories for six individuals from the MASS

The ‘Multicentre aneurysm screening study’ (MASS) (Thompson *et al*., 2009) has recorded AAA diameters, using sequential ultrasound measurements, from men aged 65–74 years for up to 11 years. In this paper data from the MASS are used to predict relevant quantities to help to inform monitoring intervals for the UK national screening programme. The MASS study is described in more detail in Section 4. However, to motivate the type of longitudinal model to use, Fig. 1 shows the observed AAA growth for six individuals from the MASS study. These individuals were chosen to illustrate the considerable variability in growth patterns between patients and the variability within growth series. Some of the variation may be explained by patients’ characteristics (e.g. age at screening, diabetes and smoking habits) (Brady *et al*., 2004) although much will remain unexplained.

A flexible model is therefore required to allow for patient-specific AAA growth that may be increasing or decreasing, linear or non-linear. Linear and quadratic hierarchical growth models can provide this flexibility and have been implemented previously to characterize AAA growth (Brady *et al*., 2004; Eriksson *et al*., 2005; Vardulaki *et al*., 1998). Hierarchical models (multilevel models) are commonly used to account for correlation in repeated measurements when data are hierarchically structured (Goldstein, 2003). Such models will be used to make predictions about the future size of aneurysm for individuals with one or more AAA measurements.

In Section 2 the linear and quadratic hierarchical growth models are introduced. In Section 3, given data for a specific individual, a variety of predictions are obtained from the model by using the estimated random effects for that individual. The MASS data set is described in more detail in Section 4, and predictions are obtained from hierarchical growth models fitted to the data. Finally we investigate extending the linear and quadratic models by relaxing the assumption of normally distributed random effects and allowing for a heavier-tailed error distribution.

## 2. Linear and quadratic growth hierarchical models

Suppose that repeated measurements of a variable are collected from *n* individuals where *y*_*ij*_ denotes the *j*th measurement from the *i*th individual, *i*=1,…,*n*, *j*=1,…,*m*_*i*_. Measurement *y*_*ij*_ is obtained at time *t*_*ij*_, where the time origin *t*=0 is well defined by, for example, a given calendar time, age or clinical measurement. Assuming a normally distributed response, and normally distributed intercepts and slopes, the basic form of the linear mixed effects growth model is


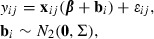
(1)

where **x**_*ij*_=(1,*t*_*ij*_) is the design vector for patient *i* at the *j*th measurement, ***β***=(*β*_0_,*β*_1_)^T^ is a vector of parameters and **b**_*i*_=(*b*_0*i*_,*b*_1*i*_)^T^ specify the individual-specific random-effects terms. The parameters *β*_0_ and *β*_1_ represent the average intercept and slope (rate of growth) respectively. The error terms *ɛ*_*ij*_ are assumed independent 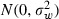, and the between-subject variance–covariance matrix Σ has variances 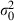 and 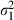 on the diagonal, and covariance *ρ*_01_*σ*_0_*σ*_1_ on the off diagonal. In addition, suppose that *p* covariates are available from individual *i* at measurement *j*, given by the *p*-dimensional vector **z**_*ij*_. If the effect of these covariates on both the intercept and the slope is of interest, then the covariate design vector **w**_*ij*_=(**z**_*ij*_,*t*_*ij*_**z**_*ij*_) can be formed to give the model


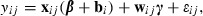
(2)

where ***γ*** is a 2*p*×1 vector of parameters containing the effect of each covariate on the intercept and slope.

The model can be extended to allow for curvature in individual growth rates by considering a quadratic growth model. The design vector then becomes 
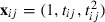
 with corresponding parameters ***β***=(*β*_0_,*β*_1_,*β*_2_)^T^, and random effects **b**_*i*_=(*b*_0*i*_,*b*_1*i*_,*b*_2*i*_)^T^ modelled by using a trivariate normal distribution. Σ is now a 3×3 variance–covariance matrix, with variances 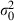, 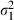 and 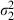, and covariances *ρ*_*kl*_*σ*_*k*_*σ*_*l*_, *k*,*l*=0,1,2, *k*≠*l*. The effect of the *p* covariates on the intercept, slope and curvature can be estimated by using the covariate design vector 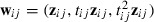 with associated 3*p*×1 vector ***γ***. We shall let *θ*=(***β***,***γ***,Σ,*σ*_*w*_) denote the vector of parameters that are to be estimated by the model.

Growth models in which the random effects are normally distributed can be fitted by using maximum likelihood or restricted maximum likelihood in various statistical packages including xtmixed (Stata) (StataCorp, 2009), gllamm (Stata) (Rabe-Hesketh *et al*., 2004), nlme (R) (Pinheiro *et al*., 2009), lme4 (R) (Bates and Maechler, 2009) and procmixed (SAS) (SAS Institute, 2008). Alternatively, a Bayesian approach can be taken where the parameters *θ* are given prior distributions, and posterior inferences are obtained via Markov chain Monte Carlo (MCMC) sampling. A thorough introduction to Bayesian hierarchical models can be found in chapter 5 of Gelman *et al*. (2003) and in Gelman and Hill (2007). Prior distributions that are specific to the AAA application are described in Section 4.2.

## 3. Hierarchical model predictions

Predictions of AAA growth can be used to inform appropriate monitoring intervals for AAA screening. Such predictions may be made from a fitted hierarchical model for possible hypothetical individuals, who are not in the original data set (an out-of-sample prediction). For example, it may be of interest to predict the diameter of an aneurysm, say 1 year after a screening measurement of 40 mm has been taken. Alternatively, a patient may have two or more repeat ultrasounds recorded, and all such measurements may then be used to make future predictions. One important question is whether the current diameter is adequate to make a precise prediction or whether repeated measurements are required.

This section deals with the situation where predictions are to be made for a specific individual given one or more response measurements with corresponding times of measurement. The predictions use random effects which have been estimated conditionally on the individual's data. Suppose that **b**_*i*_ are the random effects estimated from a linear model for individual *i* with data (**y**_*i*_,**t**_*i*_). Within a classical framework, shrunk estimates of the random effects, the empirical Bayes estimates (Verbeke and Molenberghs, 2000), can be obtained for an individual from most software packages. However, the distribution of the random effects given the observed data will generally be of a non-standard form. If, however, Bayesian MCMC sampling is used, it is easy to obtain the posterior distribution of the individual-specific random effects. Furthermore, if the cut function in WinBUGS is used (Spiegelhalter *et al*., 2003), the random effects for a new individual can be estimated without this individual's data contributing to the likelihood and updating the population parameters of the model. In what follows every prediction is a function of the parameters, random effects and possibly the measurement error.

### 3.1. Estimated rate of growth

For a linear model the rate of growth for individual *i*, 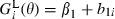, is constant over time, whereas for the quadratic model the rate of growth at time *t* is 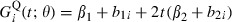. The posterior distribution for *G*_*i*_ can be easily calculated by using Bayesian MCMC sampling and inferences made from this distribution.

### 3.2. Prediction of time taken to cross a threshold given a current measurement

One prediction that may be of interest is the time taken for an individual's underlying growth curve to cross a certain threshold *α* from any given time *t*. For the linear growth model without covariates the time taken for individual *i* to hit threshold *α* can be calculated as


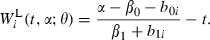
(3)

One complication with the variable 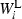 is that it may take negative values. This could happen either because the individual is already over the threshold at time *t*, or because the true growth rate is negative and hence the threshold was crossed in the past. The first case occurs when *β*_0_+*b*_0*i*_+(*β*_1_+*b*_1*i*_)*t*≥*α* and the second when *β*_1_+*b*_1*i*_<0. If, as is usual, the primary question concerns the time until the process is greater than or equal to *α*, then it is necessary to set 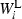 to 0 if *β*_0_+*b*_0*i*_+(*β*_1_+*b*_1*i*_)*t*≥*α*, since the process is already above the threshold. In contrast, if *β*_0_+*b*_0*i*_+(*β*_1_+*b*_1*i*_)*t*<*α* and *β*_1_+*b*_1*i*_<0 then 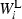 will be infinite, since the threshold will never be crossed in the future.

Using a quadratic model, predictions of this type are even more complex. For a given time *t*, interest lies in the *first* time in the future at which the threshold is crossed. As with the linear model we should first assess whether *β*_0_+*b*_0*i*_+(*β*_1_+*b*_1*i*_)*t*+(*β*_2_+*b*_2*i*_)*t*^2^≥*α*, and if so set 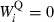. Otherwise, 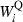 should be calculated as


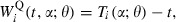
(4)

where *T*_*i*_(*α*;*θ*) is the first time *after t* at which the threshold is crossed. This can be calculated as roots to a quadratic equation:


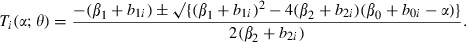
(5)

If there are no roots, then the quadratic curve will never cross the threshold, and hence 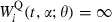 for all values of *t*. Likewise, if both roots are less than *t* then the threshold will not be crossed again, and 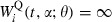. Otherwise, we take the first root of *T*_*i*_(*α*;*θ*) that occurs after *t*. Using these rules, posterior distributions for 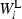 and 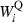 can be obtained by using Bayesian MCMC methods. In a classical analysis, the properties of this random variable are far more difficult to compute, and some simulation technique would be required.

### 3.3. Prediction of a measurement at a given future time

Predictions of future measurements can be obtained relatively easily. For the linear growth model without covariates, the predicted measurement at time *t*+*s* is simply


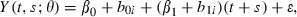
(6)

whereas for the quadratic model the estimated measurement at time *t*+*s* is


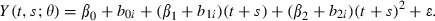
(7)

The posterior distribution of this predictive quantity is more commonly known as the posterior predictive distribution. Importantly, to obtain the probability that a measurement is above a certain value at a given future time we can simply calculate the tail area of the posterior predictive distribution corresponding to that which is above the chosen value.

## 4. The ‘Multicentre aneurysm screening study’

The MASS was set up to assess whether or not screening for AAA was beneficial in terms of long-term mortality (Thompson *et al*., 2009). Between 1997 and 1999, men aged 65–74 years were recruited from family doctor lists in four UK centres. Of the 33883 men who were invited to screening, 26875 had a visualized abdominal ultrasound scan and 1334 aneurysms (diameter 30 mm or greater) were detected. For this analysis of growth rates, data are taken from 1046 subjects who had a diameter of 30–54 mm at their first screen and at least one follow-up ultrasound measurement. The current diameter of aneurysm determined the next examination time; individuals who measured 30–44 mm were rescanned a year later, whereas those with diameters 45–54 mm were rescanned after a further 3 months. In total, the data contain 8941 ultrasound examinations. The average duration of follow-up was 4.9 years, with a mean of 8.5 ultrasound scans per person.

### 4.1. Follow-up and censoring

**Fig. 2:**
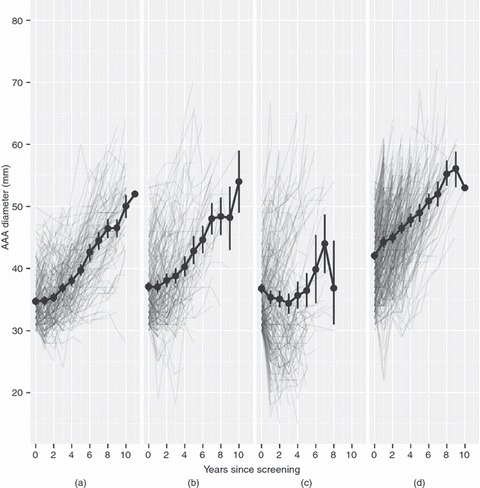
Trajectories of AAA growth given the type of censoring for all yearly AAA observations (the yearly mean AAA diameter with 95% non-parametric bootstrap confidence intervals are superimposed on the plots): (a) administrative censoring; (b) died; (c) lost to follow up; (d) surgery

Individual series are terminated either because of surgery (36%), death (21%), loss to follow-up (26%) or the administrative censoring date of March 31st, 2008 (17%), whichever comes first. Individuals whose aneurysm diameter measured 55 mm or greater at any examination or who showed rapid expansion (defined as observed growth 10 mm or more in 1 year) were considered for elective surgery. Those who were deemed unsuitable for surgery had continued surveillance of their aneurysm. A series that is terminated because the patient underwent elective surgery will tend to be biased towards a larger diameter on the final measurement due to measurement error (Brady *et al*., 2004). However, patients who drop out on the basis of their observed measurement history define a random, and hence ignorable, drop-out mechanism, if a likelihood-based analysis is used (see pages 283–285 and equation 13.2.3 of Diggle *et al*. (2002)). Fig. 2 shows four ‘spaghetti’ plots of individual growth series, grouped by the mode of termination, together with the empirical mean AAA diameter profiles. In Fig. 2 only measurements that were taken close to an anniversary of screening are used, since 3-monthly rescans were only undertaken in individuals with diameters 45–54 mm, and could distort mean values. It can clearly be seen that on average AAA diameters are larger in the group who eventually go for surgery, and those who become lost to follow-up have on average smaller AAAs. This latter observation is not entirely unexpected, since many of the measurements in the lost to follow-up group are below 30 mm, and essentially the AAA is no longer confirmed in this size range. This may explain why the patients drop out of the study. Hence there is good reason to suspect that dropout due to both surgery and lost to follow-up is mainly dependent on observed AAA diameters, and hence for this analysis we assume (missing at) random dropout.

Of the 3846 non-final ultrasounds that measured less than 45 mm, 3158 (82%) had a repeat measurement within 9–15 months, broadly following the protocol. 4041 non-final ultrasounds measured 45 mm or more, for which 2913 (72%) had a repeat measurement within 1–5 months. Appointments were therefore not always strictly adhered to, either because the patient did not attend or because the appointment was not scheduled. The effect of these missed appointments on the analysis should be minor, since these data are only intermittently missing and there is no reason to suspect that the missingness depends on unobserved AAA diameters.

### 4.2. Estimation and convergence

Both classical restricted maximum likelihood and Bayesian MCMC methods are used to obtain estimates of the parameters. Non-informative priors are used for the Bayesian models. The population mean parameters (***β***,***γ***) are given vague independent *N*(0,*τ*^2^) priors with *τ*=1000. The within-subject variance 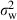 is assigned an inverse gamma prior, IG(0.001,0.001). To ensure that Σ is positive definite, an inverse Wishart prior distribution is used with degrees of freedom equal to 1 plus the dimension of Σ, i.e. 3 for the linear model, and 4 for the quadratic. This has the effect of placing a uniform distribution on each of the correlation parameters (Gelman and Hill, 2007). Inferences are based on two parallel chains, each with 10000 iterations, of which the first 500 are discarded as burn-in. The convergence diagnostic 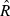 (Brooks and Gelman, 1998) is assessed for each parameter with a value close to 1 indicating good convergence properties. We obtained 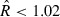 for all parameters in all models. Posterior medians (with standard deviations) from the Bayesian analyses are interpreted as equivalent to estimates (with standard errors (SEs)) from the classical analyses. The WinBUGS code for the models that are presented in this paper is available at http://www.mrc-bsu.cam.ac.uk.

### 4.3. Timescale for analysis

There are two possible choices for the timescale that is used in the longitudinal model; time since screening and age. Time since screening is relevant since at baseline the population is constrained to be within the diameters 30–54 mm; the inclusion policy of the MASS study. This is also the inclusion criterion for the UK national screening programme, and hence this timescale is highly relevant for predictions. However, using age as the timescale may be more relevant for general predictions of aneurysm growth, where the time of screening is an irrelevant quantity. A comparison of models using each timescale was first made. In a hierarchical model, the choice of timescale is important as shrinkage of the random effects can result in different estimates of mean growth and can change predictions. This is seen in Table 1, where restricted maximum likelihood estimates from classical linear and quadratic growth models, using either time since screening or age as the timescale, are presented (linear models, L1-time and L1-age; quadratic models, Q1-time and Q1-age). The estimates of mean AAA growth are quite different between the models L1-time and L1-age, and between Q1-time and Q1-age.

**Table 1:** Parameter estimates (with SEs in parentheses) from classical restricted maximum likelihood linear and quadratic growth models, using either time since screening or age as the timescale†

*Parameter*	*Estimates for the following models:*			
	
	*L1-time*	*L1-age*‡	*Q1-time*	*Q1-age*‡
*β*_0_	37.5 (0.2)	38.3 (0.3)	38.3 (0.2)	39.3 (0.3)
*β*_1_	2.19 (0.06)	1.89 (0.05)	1.48 (0.09)	1.24 (0.06)
*β*_2_	—	—	0.109 (0.009)	0.106 (0.006)
*σ*_0_	7.13	9.71	6.69	8.87
*σ*_1_	1.70	1.43	2.27	1.62
*σ*_2_	—	—	0.16	0.09
*ρ*_01_	0.52	0.34	0.58	0.67
*ρ*_02_	—	—	−0.35	−0.42
*ρ*_12_	—	—	−0.71	−0.29
*σ*_w_	3.12	3.16	2.96	3.03
log (*L*)	−25677	−26018	−25444	−25732
*k*	6	6	10	10
AIC	51366	52049	50908	51484

† Log-likelihood  log (*L*); number of parameters *k*; Akaike information criterion AIC=−2  log (*L*)+2*k*.

‡ Age centred at 70 years.

The models can be further compared by studying the Akaike information criterion AIC. Clearly a non-linear trend provides a better fit as AIC decreases dramatically in the two quadratic models. Furthermore, the use of time since screening as the timescale provides a better fit to the MASS data. In terms of prediction, AIC helps us to choose a model that will give good predictions for a new individual recruited in the same way as the sample, and clearly the models that use time since screening are better in this respect. Time since screening is therefore used as the timescale in all following models, but to make relevant predictions for the national screening programme at age 65 years we also consider including baseline age as a covariate in Section 5. This facilitates predictions to be made for a number of possible ages at screening, and in particular age 65 years.

### 4.4. Bayesian models

Table 2 shows the parameter estimates for the standard linear (L1) and quadratic (Q1) models, fitted by using Bayesian MCMC sampling. Compared with the maximum likelihood estimates that were obtained from the classical fit (Table 1), the classical and Bayesian models produce almost identical parameter estimates, suggesting that the priors that were chosen in the Bayesian models are indeed effectively non-informative. Table 2 also shows the posterior mean deviance 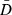, the effective number of parameters *p*_*D*_ and the deviance information criterion 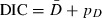 (Spiegelhalter *et al*., 2002). From model L1, the average diameter at first screen is 37.5 mm (SE 0.2), with an average growth rate of 2.2 mm year ^−1^ (SE 0.07). There is considerable between-patient variation both in AAA diameters at first screen and in growth rates, and these are positively correlated. As with the classical models, there is evidence that AAA growth is non-linear since the quadratic model (Q1) has a lower DIC.

**Table 2:** Parameter estimates from Bayesian linear and quadratic random-effects models†

*Parameter*	*Estimates for the following models:*			
	
	*L1*	*Q1*	*L2*	*L1-T*
*β*_0_	37.5 (0.2)	38.3 (0.2)	37.5‡	37.4 (0.2)
*β*_1_	2.19 (0.07)	1.49 (0.09)	2.13 (0.06)	2.18 (0.06)
*β*_2_	—	0.108 (0.012)	—	—
*σ*_0_	7.12 (0.17)	6.69 (0.16)	7.69‡	7.25 (0.17)
*σ*_1_	1.74 (0.06)	2.26 (0.10)	1.54 (0.06)	1.65 (0.06)
*σ*_2_	—	0.15 (0.01)	—	—
*ρ*_01_	0.51 (0.03)	0.58 (0.04)	0.41‡	0.51 (0.03)
*ρ*_02_	—	−0.36 (0.09)	—	—
*ρ*_12_	—	−0.71 (0.06)	—	—
*σ*_w_	3.12 (0.03)	2.97 (0.03)	3.14 (0.03)	3.25 (0.07)
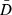	45692	44823	45810	44589
*p*_*D*_	1620	1812	1673	1723
DIC	47312	46635	47483	46312

† See Section 4.4 for a description of the models. Posterior medians with standard deviations in parentheses are shown.

‡ Empirical means and variances.

**Fig. 3:**
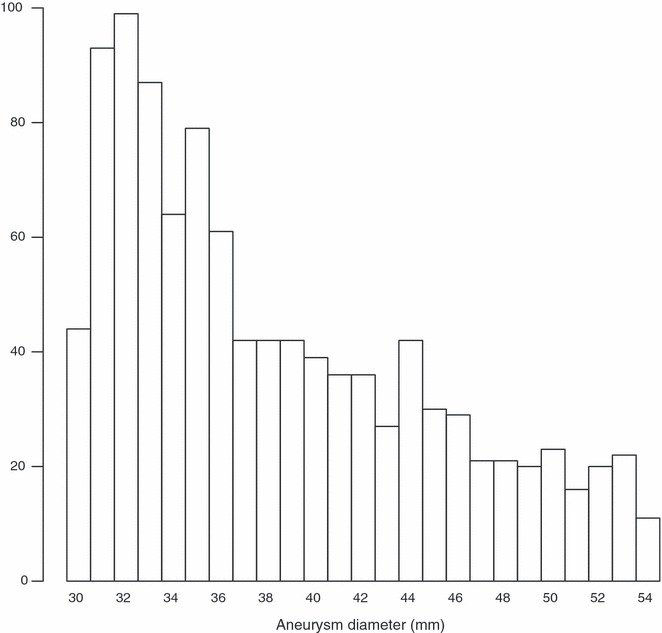
Histogram of aneurysm diameters 30–54 mm at first screen in the MASS (*n*=1046)

Fig. 3 shows the distribution of measured aneurysm diameters at first screen. Clearly the distribution is skewed and non-normal, indicating that the standard model may be inadequate. To avoid making a parametric assumption concerning the distribution of diameters at first screen, a further model (L2) allows the individual-specific intercepts to be independent, and entirely unrelated, parameters. Each individual's intercept is given an independent uniform *U*(0,1000) prior. Hence this model estimates 1046 separate intercepts with no shrinkage towards their overall mean. The random slopes are then modelled conditionally on the intercepts by assuming that the conditional distribution is Gaussian, as follows:


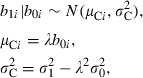
(8)

where *λ*=*ρ*_01_*σ*_1_/*σ*_0_ is given a uniform *U*(−5,5) prior. This parameterization results in *E*[*b*_1*i*_]=0 and 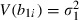. Since the population of the intercepts is not specified and hence *β*_0_ and *σ*_0_ are not parameters of the model, in Table 2 the unweighted empirical means and variances of the intercepts are presented. The standard deviation *σ*_0_ is higher than estimated in model L1, reflecting the fact that no shrinkage of the intercepts is taking place. Conversely the standard deviation of the slopes *σ*_1_ is smaller as is the empirical correlation between intercepts and slopes. Interestingly, the effective number of parameters increases by only 53 compared with the random-effects model L1 and the posterior mean deviance 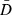 is actually higher in model L2, as is DIC. One possible reason for the increase in 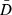 is that, since the deviance is averaged over its posterior distribution, 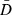 already incorporates a degree of penalty for model complexity. Indeed the relatively small increase in the effective number of parameters (compared with the 1046 individuals) suggests that there is not much shrinkage of the intercepts under model L1. Nevertheless, the smaller DIC in model L1 indicates that this is the preferred model.

There is evidence from residual plots that the within-patient variation is more heavy tailed than Gaussian. So a further model that we consider specifies a *t*-distribution for within-patient variation. The degrees of freedom of this distribution are to be estimated, and we place a *U*(2,1000) prior on the degrees-of-freedom parameter. Results from this extension to the linear model, labelled L1-T, are given in Table 2. The degrees of freedom are estimated to be close to 4, suggesting a heavy-tailed distribution, and DIC has decreased substantially compared with model L1.

**Fig. 4:**
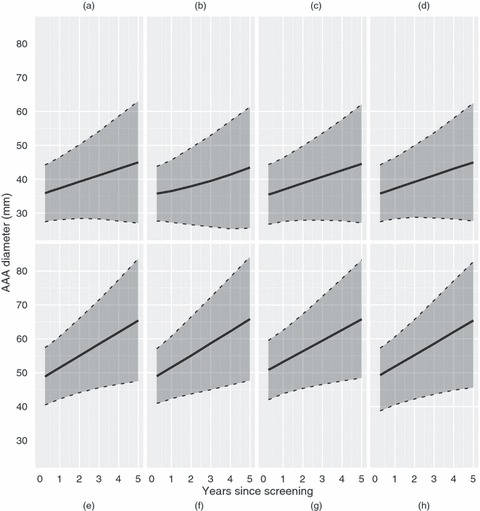
Predicted AAA diameter given a current diameter of either (a)–(d) 35 mm or (e)–(h) 50 mm taken at the time of screening, according to the four models of Table 2 (posterior medians and pointwise 95% credible intervals are presented): (a), (e) model L1; (b), (f) model Q1; (c), (g) model L2; (d), (h) model L1-T

Table 3 shows predictions for a specific individual whose AAA diameter at screening (*t*=0) is either 35 mm or 50 mm. All models estimate a similar true growth rate when *y*=35 at first screen, at approximately 2 mm year^−1^. The predicted growth rates when *y*=50 at first screen are, however, higher, at approximately 3.5 mm year^−1^. The estimated time for the underlying process to cross 55 mm is similar across all models, although the wide credible intervals limit practical usefulness of this quantity. The probabilities of crossing the 55 mm threshold within 3 months and 1 year are practically 0 for a screening diameter of 35 mm and are very similar between models L1, Q1, and L1-T for a screening diameter of 50 mm, whereas the probabilities from model L2 are higher. Fig. 4 shows predicted aneurysm growth given a single measurement at screening of either

**Table 3:** Predictions (with 95% intervals) for an individual with a single AAA diameter measurement at baseline (*t*=0) according to the four models of Table 2

*Baseline diameter (mm)*	*Prediction*	*Results for the following models:*			
		
		*L1*	*Q1*	*L2*	*L1-T*
35	*G* (mm year^−1^)	1.9	2.1	1.9	2.0
		(−1.0, 5.0)	(0.4, 5.1)	(−0.9, 4.8)	(−0.9, 4.8)
	*W*(*t*=0,*α*=55) (years)	10.1	9.1	10.3	10.1
		(3.5, ∞)	(3.7, ∞)	(3.6, ∞)	(3.6, ∞)
	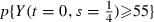	0.0	0.0	0.0	0.0
	*p*{*Y*(*t*=0,*s*=1)≥55}	0.0	0.0	0.0	0.0
50	*G* (mm year^−^1)	3.5	3.6	3.2	3.5
		(0.4, 6.5)	(1.0, 7.3)	(0.4, 6.0)	(0.5, 6.3)
	*W*(*t*=0,*α*=55) (years)	2.0	2.0	1.8	1.9
		(0.4, 39.0)	(0.4, 10.5)	(0.2, ∞)	(0.5, 23.2)
	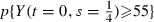	0.08	0.07	0.18	0.07
	*p*{*Y*(*t*=0,*s*=1)≥55}	0.23	0.23	0.35	0.22

- 35 mm or
- 50 mm.

In general, predictions are remarkably similar between the fitted models, with the quadratic model Q1 showing slight curvature for an individual with a diameter of 35 mm at screening. Interestingly, for an individual who measures 50 mm at screening, the predicted average AAA diameter 3 months later is slightly less than 50 mm for all models except L2. This occurs because the intercepts from all these models are shrunk towards the population mean intercept, resulting in slightly lower predictions, whereas there is no shrinkage of intercepts in model L2. By way of explanation, these random-intercept models assume that an imperfectly measured baseline diameter of 50 mm is more likely to be an outlier since it lies far from the mean baseline diameter in the population. Hence the model predicts that a subsequent measurement would on average be less than 50 mm. For an individual with a diameter of 35 mm at screening, predictions are more similar to the observed diameter since it is closer to the population mean diameter resulting in less shrinkage.

**Fig. 5:**
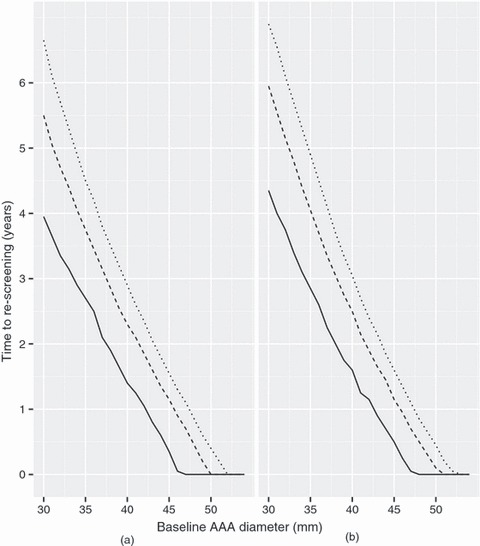
Probability of an observed AAA diameter being greater than or equal to 55 mm at rescreening given baseline AAA diameter, using (a) the first linear and (b) the quadratic models of Table 2: ⋯⋯, 10% probability; ––, 5% probability; 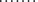, 1% probability

In terms of planning monitoring intervals for AAA, a key desire is to limit the probability that the next observation is greater than or equal to the 55 mm threshold. Such probabilities can be easily calculated from the predictive distributions in an MCMC framework, and Fig. 5 shows how these depend on the baseline AAA diameter and can be controlled by choosing the time of the next measurement. Both the linear and the quadratic models are shown in Fig. 5 for probability limits of 1%, 5% and 10%. For example, if we wish for fewer than 1% of individuals to have a diameter over the threshold at their second scan, a screening interval of 2.5 years or less would be sufficient for those who measured 35 mm at baseline. In contrast this interval would need to be 5 months or less for an individual who measured 45 mm at baseline. For individuals who measured 50 mm at baseline there is actually already a chance greater than 1% that an immediate remeasurement would result in an observed diameter that is 55 mm or more. The linear and quadratic models give very similar results.

The accuracy of the models in predicting the probability of exceeding the 55 mm threshold is investigated by forming a second, prediction, data set consisting of each individual's first *k* measurements, *k*=1,2,3. We treat each individual in the prediction data set as a new patient, independent from the analysis data set, for which new random effects are estimated. The posterior predicted probability of a measurement being greater than or equal to 55 mm is then calculated for each individual at years 1, 2, 3, 4 and 5 after screening. However, these probabilities cannot be directly compared with the observed data owing to individuals with high measurements dropping out of the study, and hence leaving a non-representative sample. Instead, multiply imputed complete data sets, as suggested by Gelman *et al*. (2005), are used as the comparator. Each multiply imputed data set is constructed as follows. For each individual at year *x* (*x*=1,…,5), the measurement and observation time closest to year *x* is used. However, if no scans are taken within 6 months of year *x* the measurement is imputed from the individual's posterior predictive distribution at year *x* (using all available data). The multiply imputed data set therefore consists of a mixture of observed data and imputed data. The percentage of missing, and hence imputed, data at years 1–5 is 3%, 10%, 23%, 34% and 49% respectively. Over all (19000 MCMC) imputed data sets the mean proportion of measurements that were 55 mm or greater was calculated as 18.5% by using model L1. This compares with predicted proportions of 17.9%, 18.4% and 18.4% when the first one, two or three scans were used for prediction respectively, suggesting an overall good predictive performance. Similar results were obtained for the other models.

### 4.5. Predictors of abdominal aortic aneurysm growth

We consider extending model L1 to include possible predictors of AAA growth. We chose to extend this model because of its simplicity and because its predictions were very similar to those of the more complex models. At first repeat scan individuals were asked about their current smoking habits. 97 individuals reported never smoking compared with 585 previous smokers and 317 current smokers. Smoking data were missing for 47 individuals. The population parameters for this model are very similar to those for model L1 although there is strong evidence that previous and current smokers have on average larger diameters at baseline than non-smokers, by 2.4 mm (SE 0.8) and 2.5 mm (SE 0.9) respectively, and faster growth than non-smokers, by 0.53 mm year^−1^ (SE 0.21) and 0.82 mm year^−1^(SE 0.22) respectively. The age of an individual at baseline was also considered as a predictor of aneurysm growth. There was found to be no evidence of an association between age and AAA size at screening (−0.08 mm per year of baseline age; SE 0.08), and only a small association between age and the rate of AAA growth (−0.04 mm year^−1^ per year of baseline age; SE 0.02). The surprising negative coefficient, suggesting smaller AAA growth in the older population, may be due to the MASS selection process. One hypothesis is that fast growers in the older population will have diameters that are too large to be included in the MASS, whereas slow growers in the young population have diameters that are too small for selection. Such a selection bias could produce an apparently negative association between age and growth.

**Fig. 6:**
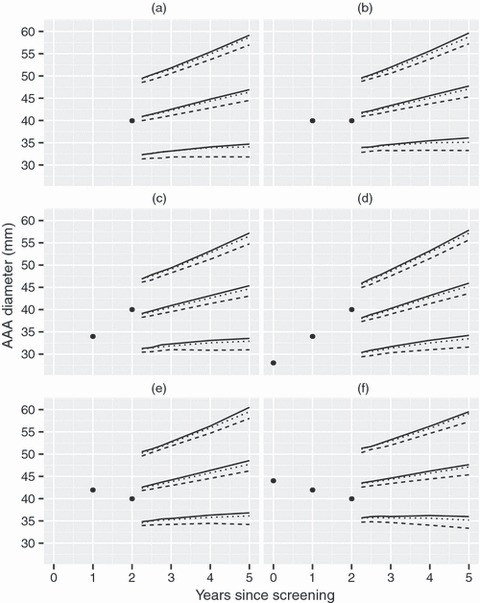
Predicted AAA diameter for an individual aged 65 years at screening, by smoking status and observed growth (posterior medians and pointwise 95% credible intervals are presented for each prediction; see Section 4.5 for explanation): 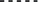, current smoker; ⋯⋯, ex-smoker; ––, non-smoker

Fig. 6 shows how predictions vary depending on the number and pattern of previous observations and the smoking status of an individual. All predictions are shown for individuals aged 65 years at screening who have a 40-mm-diameter aneurysm observed 2 years after screening. Fig. 6(a) is based on a single 40-mm-diameter measurement taken 2 years after screening and can be used as the reference prediction. Fig. 6(b) presents an individual with two 40 mm measurements at *t*=1 and *t*=2, and predictions are slightly higher in this scenario. Figs 6(c) and 6(d) show predictions for ‘fast growers’ who have observed growth rates of 6 mm year^−1^ (approximately 2 standard deviations above the population mean). Meanwhile, Figs 6(e) and 6(f) show predictions for individuals whose AAA is observed to ‘shrink’ at a rate of −2 mm year^−1^ (approximately 2 standard deviations below the population mean). Predictions change only very slightly between smoking categories, despite the highly significant effect of including this variable as a covariate in the model. In contrast, previously observed AAA diameters do alter predictions, suggesting that the whole history is important, not just the final diameter. Since the average observed diameter for individuals who ‘shrink’ (Figs 6(e) and 6(f)) is greater than that for the ‘fast growers’ (Figs 6(c) and 6(d)), predictions are actually higher for these individuals. Surprisingly, the number of measurements does not apparently alter the precision of the predicted diameter, although the predicted size of AAA does change slightly between patients who have two measurements compared with those who have three. Finally, all the predicted growth curves appear to pass close to the average observed diameter at the average observation time.

## 5. Discussion

We have shown in this paper how various predictions can be made by using a linear or quadratic hierarchical mixed effects model. Two novel aspects arise from this work. Firstly, we have extended the mixed effects models to incorporate error and random-effects distributions that are non-normal, within a Bayesian framework. Secondly, we make predictions for a specific individual by using random effects estimated conditionally on their data.

A slightly different prediction approach has been described by Skrondal and Rabe-Hesketh (2009). Here, the population-averaged predicted mean response is obtained analytically by integrating over the random-effects distribution, whereas uncertainty in the mean response is addressed by simulating the parameters from their sampling distribution. A parametric bootstrap procedure has also been proposed as a way of obtaining a prediction interval for the mean response given values of the covariates (Hall and Maiti, 2006). Meanwhile, Taylor and Law (1998) have given an account of how in-sample predictions of future observations can be obtained from closed form solutions when multivariate normality is assumed.

One practical advantage of using Bayesian modelling within a flexible software package such as WinBUGS is that many different model extensions can easily be investigated. For example, it is clear that for an aneurysm-detected population the baseline distribution of aneurysm diameters is non-normal. We have tried to relax the normality assumption placed on our random effects by fitting an independent intercepts model (L2), although we could have investigated other parametric distributions. An alternative approach is to model more precisely the process by which the data were obtained. Specifically, individuals who were screened and deemed ‘normal’, i.e. had a diameter less than 30 mm, were not followed up further and hence were excluded from the analysis data set. Therefore in reality, in addition to the 1046 measured AAA diameters at first screen, there are also 25541 left-censored diameters, in which we know only that *y*<30. Each of these individuals have the following likelihood contribution:


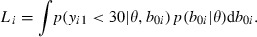
(9)

By adding these contributions to the likelihood, our inferences are then about the general population, and not specifically about those with an aneurysm. As a consequence, for all individuals with a detected aneurysm, their estimated baseline diameters are likely to be shrunk downwards towards the population mean. For example, an individual whose observed diameter is 30 mm at first screen is more likely to have their true diameter less than 30 mm, owing to our knowledge that the population mean aneurysm diameter is far smaller. This behaviour can only be modelled if the censoring mechanism is fully incorporated. We have attempted to fit censoring models by assuming that the population distribution of AAA diameters at first screen follows either a Gaussian distribution or *t*-distribution with the degrees of freedom estimated by the model. The population mean diameter at first screen was estimated to be close to 20 mm whereas the population mean rate of growth was 0.05 mm year^−1^ for the model with Gaussian intercepts, and 1.18 mm year^−1^ for the model with *t*-distributed intercepts. Hence, estimates from these models are highly sensitive to the choice of distribution for the intercepts. This is because 95% of the individuals are censored and measurements for only the upper 5% tail of the distribution are available. To our knowledge, the issue of such censoring or truncation has been rarely addressed when using longitudinal mixed effects models. Mehrotra *et al*. (2000) proposed an EM-like algorithm to obtain maximum likelihood estimates when the sample is truncated, but only for a fixed subject effects model. Further investigation into the behaviour of mixed effects models when censoring or truncation is present would therefore be of interest.

The non-linear growth of AAAs has been shown previously (Brady *et al*., 2004; Vardulaki *et al*., 1998). The use of mixed effects models with correlated intercept and linear growth rates allows individuals with higher baseline measurements to have faster growth. Accelerated growth within an individual's growth series can also be modelled by using a non-linear model. However, we have shown that using either a quadratic or a linear model gave remarkably similar predictions over the time period of interest for AAA monitoring. Since difficulties are associated with making predictions from a quadratic model, we question the practical relevance of this model for this application. Indeed quadratic growth may be unrealistic in the long term, with predictions possibly showing a reversal in the direction of growth for some individuals. The linear model, despite representing a simplified version of the true nature of AAA growth, appears to be adequate for short-term predictions.

In all the models fitted there was found to be very substantial between-individual variation, which requires further exploration. The baseline smoking status of an individual was found to be significantly associated with both baseline AAA diameter and the rate of growth. Nevertheless, the between-individual standard deviations for the intercept and slope decreased by only 0.4% as a result of adding smoking status as a covariate. Other variables that have been shown to correlate with AAA growth rates include diabetes and atherosclerosis (Brady *et al*., 2004); although such variables could be included in future models, it is unlikely that they would impact importantly on relevant predictions.

A variety of predictions can be made from longitudinal models, such as the time to reaching a certain threshold, or the predicted level of the observed or underlying outcome after a given time period. In our AAA application, however, we find that a prediction of the time taken to reach a threshold diameter of 55 mm is of little practical use, since the prediction is very imprecise. This has been noted previously in relation to time-to-event predictions in the context of survival analysis (Graf *et al*., 1999). Joint longitudinal data and survival modelling (Tsiatis *et al*., 1995) is inappropriate in our application, since we are modelling the time to an underlying threshold that is an aspect of the longitudinal process, rather than to an observed event. More relevant for planning monitoring intervals is the distribution of the (observed) outcome after a given time, and the probability that a future observation will be greater than a specified threshold at that time. Expressing the prediction in terms of the probability of crossing the threshold provides a rational basis for planning appropriate monitoring intervals.
